# Supplementary material for: Role of Nrf2 Nucleus Translocation in Beauvericin-Induced Cell Damage in Rat Hepatocytes
Source: Toxins (Basel). 2022 May 25;14(6):367. doi: 10.3390/toxins14060367 (PMC9229947; doi:10.3390/toxins14060367)
Supplement: Supplementary file 1 [file toxins-14-00367-s001.zip › toxins-1741730-supplementary.pdf]

toxins

Article

Role of Nrf2 Nucleus Translocation in Beauvericin-Induced Cell Damage in Rat Hepatocytes

Jiabin Shi, Yaling Wang, Wenlin Xu, Guodong Cai, Hui Zou, Jianhong Gu, Yan Yuan, Zongping Liu and Jianchun Bian

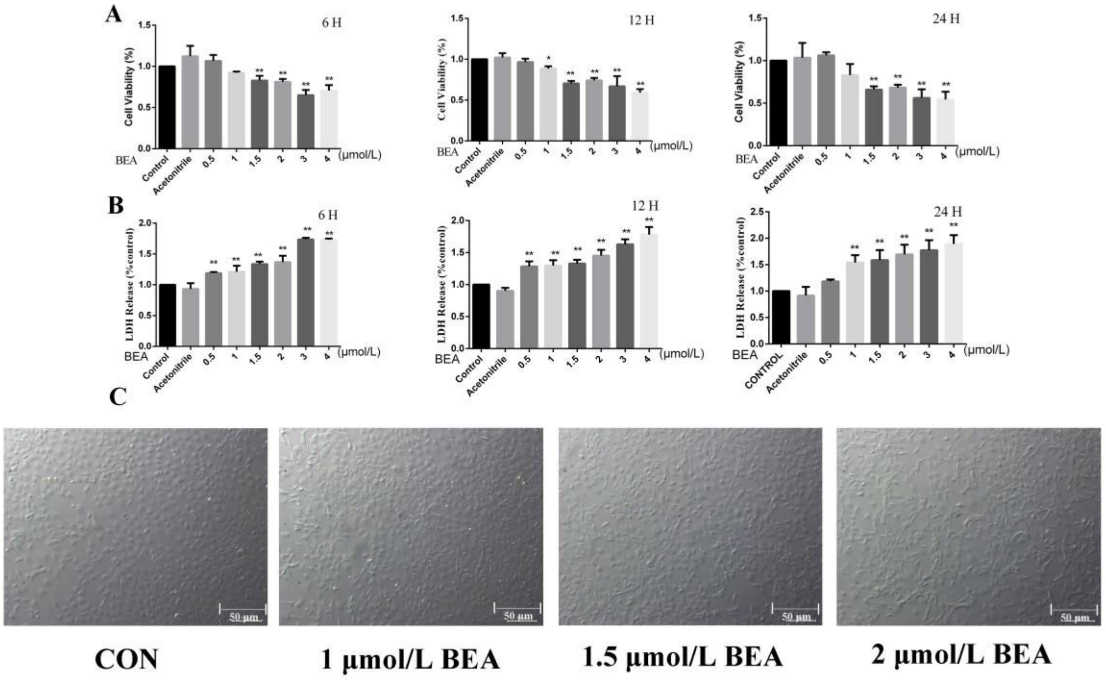

Figure S1. Liver cell injury induced by BEA. (**A**) BRL3A cells were treated with BEA at different concentrations of 0, 0.5, 1, 1.5, 2, 3, and 4 μmol/L for 6, 12 and 24 h. The acetonitrile group was added to determine whether the solvent was cytotoxic. CCK8 method was used to detect the effect of BEA on the viability of BRL3A cells. (**B**) The release of LDH was measured by LDH release assay kit to discover BEA-induced cytotoxicity. All experiments were performed in triplicate (n=3) and data were presented as the means ± SD and analyzed by one-way ANOVA. * $p < 0.05$ and ** $p < 0.01$, compared with the blank control group (CON). (**C**) Morphology of BRL3A cells after exposure to 0, 1, 1.5 and 2 μmol/L BEA for 12 h were observed using an optical microscope. Scale bar = 50 μm.

Table S1. The primary and secondary antibodies used in the present study.

Antibody name	Dilution ratio	Manufacturers
Bax	1:1000	Abcam (Waltham, MA, USA)
Bcl-2	1:1000	Abcam (Waltham, MA, USA)
Cleaved-caspase 3	1:1000	Proteintech Group, Inc (Wuhan, China)
Cleaved-caspase 9	1:1000	Proteintech Group, Inc (Wuhan, China)
Nrf2	1:1000	Cell Signaling Technology (Beverly, MA, USA)
Keap 1	1:1000	Santa Cruz Biotechnology, Inc (Dallas, Texas, USA)
NQO1	1:1000	Abclonal Technology (Wuhan, China)
HO-1	1:1000	Cell Signaling Technology (Beverly, MA, USA)
GST	1:1000	Cell Signaling Technology (Beverly, MA, USA)
p62	1:1000	Abclonal Technology (Wuhan, China)

Beclin1	1:1000	Cell Signaling Technology (Beverly, MA, USA)
ATG5	1:1000	Abcam (Waltham, MA, USA)
LC3	1:1000	Abclonal Technology (Wuhan, China)
Histone H3	1:2000	Cell Signaling Technology (Beverly, MA, USA)
GAPDH	1:20000	Proteintech Group, Inc (Wuhan, China)
β-actin	1:5000	Proteintech Group, Inc (Wuhan, China)
Peroxidase conjugated secondary antibody	1:10000	Jackson ImmunoResearch Inc (Langcaster, PA, USA)

Table S2. Sequence of primers for real-time RT-PCR amplification.

**Primers**	**Primer Sequences**	**Product Length (bp)**
NQO1	Forward: 5′- CAGACCTGGTGATATTTCAGTTCC -3′<br>Reverse: 5′- CACCCTGCAGAGAGTACATGG -3	107
HO-1	Forward: 5′- GAGCCAGCCTGAACTAGC -3′<br>Reverse: 5′- GATGTGCACCTCCTTGGT -3′	198
SOD	Forward: 5′- ATTCACTTCGAGCAGAAGGCA -3′<br>Reverse: 5′- TGAGGTCCTGCAGTGGTACA -3′	137
GST	Forward: 5′- AGCTATGCCACCGTACACC -3′ Reverse:<br>5′- GAGCTGCCCATACAGACAAGTG -3′	163
Beclin 1	Forward: 5′- TCGGGGCCTAAAGAATGGAG -3′<br>Reverse: 5′- GCCTGGGCTGTGGTAAGTAA -3′	166
P62	Forward: 5′- TTTCTCGGATGAAGGCGGCT -3′<br>Reverse: 5′- ACAAGGGAGGTGGGTTGTGG -3′	147
LC3 B	Forward: 5′- AAGCCAACACAGCCACCTCT -3′<br>Reverse: 5′- CTTCCCGACCGCACCATAGT -3′	174
β-actin	Forward: 5′- TCACCCACACTGTGCCCATCTATGA -3′<br>Reverse: 5′- CATCGGAACCGCTCATTGCCGATAG -3′	295

Sequence of primers for real-time RT-PCR amplification.

Table S3. Procedures of real-time qRT-PCR amplification.

**Procedure**	**Temperature**	**Time**	**Cycle number**
Pre-denaturation	95 °C	5 min	1
Denaturation	95 °C	10 sec	
Annealing	55–60 °C	20 sec	40
Extension	72 °C	20 sec	

Procedures of real-time qRT-PCR amplification.
